# The SHINE Trial Infant Feeding Intervention: Pilot Study of Effects on Maternal Learning and Infant Diet Quality in Rural Zimbabwe

**DOI:** 10.1093/cid/civ846

**Published:** 2015-11-11

**Authors:** Amy Desai, Laura E. Smith, Mduduzi N. N. Mbuya, Ancikaria Chigumira, Dadirai Fundira, Naume V. Tavengwa, Thokozile R. Malaba, Florence D. Majo, Jean H. Humphrey, Rebecca J. Stoltzfus

**Affiliations:** 1 Department of International Health, The Johns Hopkins Bloomberg School of Public Health, Baltimore, Maryland; 2 Division of Nutritional Sciences, Cornell University, Ithaca, New York; 3 Zvitambo Institute for Maternal and Child Health Research; 4 Ministry of Health and Child Care, Harare, Zimbabwe

**Keywords:** infant and young child feeding, intervention design research, dietary assessment, complementary feeding, lipid-based nutrient supplement

## Abstract

The Sanitation Hygiene Infant Nutrition Efficacy (SHINE) trial is designed to measure the independent and combined effects of improved water, sanitation, and hygiene and improved infant feeding on child stunting and anemia in Zimbabwe. We developed and pilot-tested the infant feeding intervention delivered by 9 village health workers to 19 mothers of infants aged 7–12 months. Between September 2010 and January 2011, maternal knowledge was assessed using mixed methods, and infant nutrient intakes were assessed by 24-hour recall. We observed positive shifts in mothers' knowledge. At baseline, 63% of infants met their energy requirement and most did not receive enough folate, zinc, or calcium; none met their iron requirement. Postintervention, all infants received sufficient fat and vitamin A, and most consumed enough daily energy (79%), protein (95%), calcium (89%), zinc (89%), folate (68%), and iron (68%). The SHINE trial infant feeding intervention led to significant short-term improvements in maternal learning and infant nutrient intakes.

Early childhood undernutrition causes 45% of child deaths [1] and leads to long-term cognitive deficits, fewer years and poorer performance in school, lower adult economic productivity, and a higher risk that one's children will also be undernourished, perpetuating the problem into future generations [2]. The complementary feeding period, when solid foods are introduced to complement breast milk, is a high-risk period for growth failure [3]. Inadequate nutrient density of foods and inappropriate feeding practices from 6 to 24 months of age [4] are common problems and priorities for intervention during this period of greatest vulnerability [5].

In our previously published formative research [6], we conducted Trials of Improved Practices (TIPs) [7] with 40 mothers of infants aged 6–12 months to assess the feasibility of improving infant diets using locally available foods alone or in combination with a small-quantity lipid-based supplement (SQ-LNS) in rural Zimbabwe. Poor dietary diversity and low energy density were the most common infant feeding problems. Two series of TIPs counseling sessions were conducted by professional nutritionists, and changes in infant diet quality were assessed by pre- and postcounseling 24-hour diet histories. In the first series of sessions, mothers were taught to process a wide variety of family foods and add them to their infant's thick porridge. In a second series of sessions, mothers were counseled to add SQ-LNS in addition to local foods to thick porridge. Dietary intakes of energy, protein, vitamin A, folate, calcium, iron, and zinc from complementary foods increased significantly after counseling, with or without the provision of SQ-LNS (*P* < .05). Postintervention, mothers fed infant diets that were nutritionally adequate in all nutrients except iron and zinc using local foods alone; the addition of SQ-LNS closed the iron and zinc gaps. Importantly, this study demonstrated the importance of mothers experiencing “transformational learning”—learning which goes beyond simply receiving knowledge but rather changes what one believes or understands as a result of the learning [6].

The goal of the present pilot study was to build on learning from the TIPs to design and pilot an infant and young child feeding (IYCF) intervention for implementation in the Sanitation Hygiene Infant Nutrition Efficacy (SHINE) trial. Across all 4 treatment arms, village health workers (VHWs) make 15 visits to SHINE mother–infant dyads when they deliver specified primary healthcare messages. Four of these visits focus on promotion of exclusive breastfeeding (EBF); that is, EBF is part of SHINE's standard of care equally promoted through all 4 treatment arms. During some of these 15 visits, VHWs in WASH, IYCF, and WASH +IYCF clusters also deliver lessons specific to the randomized intervention. Both the WASH and IYCF interventions (the randomly allocated interventions) are comprised of 5 core modules, each focused on a key message and delivered to the mother at a specified relevant fetal or infant age. The biologic objective of the IYCF intervention is to optimize nutritional adequacy of the infant's diet from 6 to 18 months, following 6 months of EBF. All appendices are available electronically as supplementary material, including supplementary tables and intervention modules.

## DESIGNING THE SHINE TRIAL INFANT FEEDING INTERVENTION

Based on the TIPS findings, we designed an IYCF intervention to focus on increasing nutrient density, increasing dietary diversity, communicating the importance of locally available foods for infant health, and the introduction of SQ-LNS (nutrient composition is included in Table 1 of Supplementary Appendix). In addition, other formative work within SHINE [8] had shown that parents value health, growth, and intelligence in their children, so we included the link between optimal IYCF and these child qualities. Finally, we included responsive feeding and the importance of continued breastfeeding and increased feeding during and after illness, as these care practices are often lacking throughout the world and can augment the risk of malnutrition [4].

A draft intervention was designed as 5 core modules to deliver these key messages to mothers sequentially (Table 1; full modules and tools are included in the Supplementary Appendix, Documents 1 and 2). Each module builds on the concepts covered in previous modules to reinforce learning. The modules were designed to be delivered at specific infant ages (from 5 months to 9 months) to allow the mother to accumulate new practices and incorporate them into her daily infant feeding practices without overwhelming her with too many messages at one time. Also, the modules and training of VHWs were informed by principles of adult learning [9]. Each module included tools and/or activities to illustrate key concepts—for example, an interactive puzzle to demonstrate the finite small volume of an infant stomach. During the lesson on the message “a child can eat anything an adult eats if it is processed so an infant can swallow it,” the VHW prepares any food that the mother has on hand, demonstrating how to chop or mash it and mix it into thick porridge; the mother then feeds the food to her infant as part of the lesson. The dietary diversity lesson includes a puzzle of an infant bowl; pieces are foods shaped according to food group, such that making a complete meal requires including a food from each group.

**Table 1.** Infant and Young Child Feeding Behavior Change Intervention Modules (Messages and Material) Inputs to Optimize Adequacy of Dietary Intake by Infants, Sanitation Hygiene Infant Nutrition Efficacy (SHINE) Trial

Timing of Intervention	Material Inputs	Key Behavioral Message
5 mo	None	Giving your baby the right food and liquids will help him/her to grow healthy and strong; fight infections; develop good intelligence. Continue exclusively breastfeeding until 6 mo, then give other foods in addition to breast milk.
6 mo	LiNS formulation^a^	Prepare and feed your baby thick porridge and 20 g of LiNS daily.
7 mo	LiNS formulation^a^	Your baby can eat anything an adult can eat if you process foods and mix them into porridge so your baby can swallow it.
8 mo	LiNS formulation^a^	If your baby falls ill: breastfeed more frequently and feed more food to your baby during and after illness to quicken recovery.
9 mo	LiNS formulation^a^	Feed your baby foods from each food group at every meal to make sure that he/she is receiving all the nutrients needed for healthy growth.

Abbreviation: LiNS, lipid-based nutrient supplement.

^a^ Formulation used as described by Arimond et al [19]. See Table 1 in Supplementary Appendix.

### Study Population

The draft intervention was pilot-tested in Gweru district, which is adjacent to the SHINE trial districts, between September 2011 and January 2012. The objectives of the pilot were to test understanding of the messages and tools and feasibility of delivering the intervention by VHWs. Accordingly, in consultation with the Ministry of Health and Child Care district health staff, 9 VHWs were selected to participate in the pilot study. A sample of 19 mothers who had infants 7–12 months of age were selected from registers maintained by the selected VHWs. Approximately 20 mothers had been adequate to reach saturation in previous qualitative studies on infant feeding in rural Zimbabwe [6, 10]. The pilot intervention was delivered by 9 VHWs who were trained for 2 weeks prior to the intervention. Training included communication, adult learning techniques, knowledge of messages specific to the intervention, and utilization of interactive tools to deliver the intervention.

### Data Collection

Mothers were visited in their home by a research assistant. Following written informed consent, the research assistant conducted a semistructured interview and administered a structured questionnaire to assess the mother's knowledge, attitudes, and practices regarding infant feeding and a 24-hour recall of the infant's dietary intake with quantitative food measurement [6]. On the following day, the VHW delivered module 1 of the draft IYCF intervention. One week later, the enumerator visited the mother again to assess understanding and uptake of the practices promoted in module 1 through a semistructured interview and quantitative 24-hour recall of the infant's diet. On the following day, the VHW delivered module 2 of the draft IYCF intervention; the enumerator assessed understanding and uptake of module 2 one week later. This process continued through delivery and assessment of module 5. All semistructured interviews were audio-recorded.

### Recall Method and Analysis

A combination of food composition databases was used [11–13]. For multicomponent dishes, the recipes for the dishes were collected, entered into Nutrisurvey (World Health Organization [WHO]), and nutrient intake calculated from the portion that the child had consumed. Nutrient intakes and nutrient densities were calculated from 24-hour dietary recall data and reported as median and interquartile range because the majority of the results were not normally distributed. Nutrient densities were reported as densities per 100 kJ of food consumed per day. To assess differences in intake across the 7 time points, results that were not normally distributed were transformed, and repeated-measures analysis of variance was conducted with pairwise comparisons. *P* values <.05 were considered statistically significant. To assess diet adequacy, energy and nutrient intakes were compared with the estimated requirement using the WHO estimated energy requirement, the US Department of Agriculture–recommended daily allowance for protein, and the WHO-recommended nutrient intakes (RNIs) for vitamins and minerals [4, 14–16]. Required nutrient intake from complementary foods was calculated by subtracting the amount of energy/nutrient in an average intake of breast milk (600 g for 6–8 months, 550 g for 9–11 months, and 500 g for 12–23 months) from the total requirement [17]. The estimated energy requirement was calculated as kilojoules required per kilogram of body weight using median standardized bodyweight for each category [17]. The requirement for fat was estimated to be 30% of the energy requirement [17]. The estimated requirement from complementary food for calcium was calculated using a recommendation between the US dietary reference intakes and the WHO RNIs because of the large discrepancy between the 2 reference values [18]. For zinc and iron, 30% and 10% bioavailability, respectively, was assumed [17]. The final values for nutrient requirements by infant age category are listed in Table 2 in the Supplementary Appendix. For each nutrient, the percentage of children meeting the requirement from complementary food was reported.

### Analysis of In-depth Interviews

Audio-recorded interviews were transcribed and translated into English. A deductive analysis of the transcripts was conducted to identify and code learning statements relevant to information communicated through the intervention. Coded data were categorized as knowledge recall, transformational learning, and knowledge gaps. Data from the knowledge questionnaires were tabulated to compare baseline and follow-up responses.

All analyses were conducted with Stata software version 12. Ethical approval to conduct this study was granted by the Medical Research Council of Zimbabwe and The Johns Hopkins School of Public Health Institutional Review Board.

## RESULTS

### Baseline Knowledge of Infant Feeding Practices

At baseline, most mothers knew that infant feeding was important, but either had incorrect information or not enough information to practically improve their infant's diet. For example, of the 19 mothers, 16 thought infants should be fed thin rather than thick porridge; none of the 19 mothers could describe how to feed a child recovering from illness; most mothers knew that “dietary diversity” was important, but none could list a variety of foods that could be fed to an infant.

### Baseline Infant Dietary Intake

At baseline, none of the infant diets were nutritionally adequate based on the 24-hour recall (Table 2). Nutrient densities at baseline were below recommendations [17] for vitamin A, folate, calcium, iron, and zinc, with iron being the most pronounced at less than a tenth of the recommended density (Figure 1 in Supplementary Appendix).

**Table 2.** Median Nutrient Intakes and Proportion Meeting Requirements^a^ at Baseline and After Sequential Complementary Feeding Modules (n = 19 Rural Zimbabwean Infants)

Nutrient	Baseline	FU1: After Exclusive Breastfeeding Module	FU2: After Feed Thick Porridge/SQ-LNS Module	FU3: After Babies Can Eat Anything Adults Can Eat Module	FU4: After Feeding Child During Illness Module	FU5: After Feed All 4 Food Groups Module	FU6: After Review Module
Child age, mo, mean (range)	9.1 (6.5–11.9)	9.5 (6.9–12.1)	10.5 (8.3–13.4)	11.0 (8.8–13.7)	11.5 (9.0–13.9)	11.7 (9.1–14.1)	12.0 (10.1–14.4)
Energy							
kJ/day	1759^b^ (1020, 2785)	1887^b^ (1069, 2761)	2508^b,c^ (1510, 2745)	2379^c^ (1992, 3341)	2745^c^ (1749, 3266)	2447^c^ (2111, 3310)	2635^c^ (1957, 3325)
% Met Req	63	68	68	79	79	95	79
Protein							
g/day	9.9^b^ (5.2, 12.7)	8.6^b^ (4.4, 11.8)	11.4^b,c^ (6.4, 13.8)	16.7^d^ (10.0, 23.5)	13.5^d^ (8.1, 19.8)	13.1^d^ (10.1, 18.8)	15.8^c,d^ (10.9, 17.4)
% Met Req	84	84	100	95	100	100	95
Fat							
g/day	9.3^b^ (6.0, 17.7)	10.4^b,c^ (6.4, 19.2)	16.1^c,d^ (13.5, 21.0)	19.1^d^ (13.3, 25.0)	16.7^d^ (13.7, 21.9)	18.0^c,d^ (12.8, 23.9)	20.5^d^ (15.5, 23.4)
% Met Req	89	84	84	89	100	95	100
Vitamin A							
µg RAE/day	58.0^b^ (0.8, 238.2)	122.8^b^ (2.3, 216.9)	498.9^c^ (403.7, 522.2)	555.8^c,d^ (447.4, 648.7)	555.8^d^ (477.4, 731.1)	571.5^d^ (477.6, 782.1)	678.0^d^ (557.6, 808.8)
% Met Req	79	84	89	100	100	100	100
Folate							
µg/day	26.4^b^ (10.7, 52.4)	31.9^b^ (13.7, 64.0)	107.4^c^ (86.0, 131.5)	120.4^c^ (87.5, 150.2)	104.4^c^ (96.8, 136.8)	102.1^c^ (93.2, 122.1)	119.5^c^ (99.0, 136.1)
% Met Req	31	37	84	74	74	58	68
Calcium							
mg/day	58.7^b^ (28.0, 85.0)	72.3^b^ (35.1, 167.0)	326.1^c^ (307.1, 380.8)	326.3^c^ (294.5, 440.3)	330.1^c^ (314.5, 376.6)	336.2^c^ (251.8, 363.8)	352.6^c^ (321.1, 419.6)
% Met Req	10	16	89	84	95	79	89
Iron							
mg/day	2.4^b^ (1.1, 3.3)	2.6^b^ (1.6, 3.4)	8.6^c^ (6.8, 9.6)	8.9^c^ (6.7, 10.4)	8.2^c^ (7.5, 9.8)	7.5^c^ (5.7, 9.4)	8.9^c^ (7.1, 9.8)
% Met Req	0	0	37	63	63	58	68
Zinc							
mg/day	2.3^b^ (1.4, 3.3)	2.5^b^ (1.2, 3.3)	10.0^c,d^ (8.7, 10.8)	10.4^c,d^ (9.0, 12.7)	10.6^c^ (9.4, 12.0)	7.5^d^ (5.4, 11.1)	11.0^c,d^ (9.6, 11.7)
% Met Req	16	21	89	95	100	89	89

Data are presented as median (interquartile range) unless otherwise specified. Cells without a shared letter (eg, b,c,d) denote a statistically significant difference with a *P* value <.05.

Abbreviations: FU, follow-up; Met Req, met daily requirement; RAE, retinol activity equivalent; SQ-LNS, small-quantity lipid-based supplement.

^a^ World Health Organization (WHO) estimated energy requirement, the US Department of Agriculture–recommended daily allowance for protein, and the WHO-recommended nutrient intakes for vitamins and minerals [4, 14–16].

### Postintervention Changes in Maternal Knowledge and Behavior

Maternal knowledge about infant feeding increased after receiving each of the modules (Table 3 in Supplementary Appendix). Responses during semistructured interviews reflected transformational learning on key feeding behaviors:

It is believed that thin porridge is easy for baby to swallow, since his throat is small. The elders were misleading us to give thin porridge. From the lesson you realize that baby can swallow thick porridge, it is digestible, and [the baby] passes stool normally.

It has always been said, “do not give eggs to the baby because his teeth will not come out and eggs cause fits,” therefore I had not given him some [eggs]. Even when we have fish, we gave him the broth not knowing that if you shred it [the fish], he will be able to eat. Even with vegetables, we have been giving him the broth only because we did not know [he could eat the vegetables]. Now I know how to prepare these foods and give them to the baby.

The information awakens us as mothers to care for our babies. I liked the module because at times when the baby refuses to eat food, you can just give up on trying to feed him. But now I know that the baby can refuse to eat at times, and as a mother it is up to me to try giving the baby food many times and even more so when he is sick because he could lose weight. If I feed him, he is likely to regain his weight.

### Postintervention Changes in Infant Nutrient Intakes

After the 5 modules, there were significant increases in the quantities of each nutrient consumed (Table 2). Additionally, all infants received sufficient fat and vitamin A, and a majority consumed enough daily energy (79%), protein (95%), calcium (89%), and zinc (89%). The percentage of infants meeting their folate requirement more than doubled but only reached 68%, and infants meeting their iron requirement increased from 0% to 68%.

The pattern of changes in dietary intake over the course of the 5 modules (Table 2) suggest that both the supplementation with SQ-LNS (module 2) and the educational messages about feeding a more diverse diet (module 3) contributed to increases in infant nutrient intake. Infant dietary intakes of fat and every micronutrient measured rose significantly after module 2 introducing SQ-LNS and thick porridge. However, intakes of energy and protein increased significantly after module 3. These results indicate that dietary quality improved as both the quantity and density of nutrient intakes significantly increased. Nutrient densities for all nutrients significantly increased across the time period, with the largest increases attributable to the addition of SQ-LNS at the second module (Figure 1 in Supplementary Appendix).

### The SHINE IYCF Intervention

In finalizing the SHINE Trial IYCF intervention, we chose to use an updated formulation of the lipid-based nutrient supplement developed by the International Lipid-Based Nutrient Supplements (iLiNS) Project (the iLiNS Formulation) to more closely meet the requirements of children in the 3 African countries where that project was conducted [19] (Table 1 in Supplementary Appendix). The final intervention sequenced delivery of 5 key messages among infants aged 5–9 months (Table 1). SQ-LNS is delivered to IYCF households monthly.

## DISCUSSION

Receipt of the draft IYCF intervention by 19 mothers participating in this pilot evaluation was associated with increased maternal knowledge and evidence of transformational learning. Most importantly, based on repeated 24-hour recalls of the infants' diets, nutritional adequacy improved significantly.

Improvements in infant dietary intake observed in this pilot evaluation in which VHWs delivered modules were similar in magnitude to improvements observed when the counseling interactions were provided by trained district nutritionists in our previous study [6]. In both studies, mothers fed substantially improved infant diets using local foods, but SQ-LNS was required to close key nutrient gaps, especially iron, zinc, and calcium. After all modules were completed, the nutrient gaps that most frequently remained were of folate and iron.

In spite of dietary iron gaps remaining for about one-third of the infants, the SQ-LNS formulation used in the SHINE trial contains 6 mg per daily dose, rather than the 9 mg used in this pilot. This decision was taken in line with the iLiNS formulation to mitigate concerns about the potential effect of supplemental iron on infections; there is evidence that iron supplementation in iron-sufficient children may increase risk of malaria infection, diarrhea, and other related infections [19–22]. Additionally the SQ-LNS formulation used in the SHINE Trial has increased levels of energy, fat, linoleic acid, α-linoleic acid, zinc, calcium, phosphorus, potassium, magnesium, selenium, and manganese in line with the iLiNS formulation [19] (Table 1 in Supplementary Appendix).

Two limitations of this study deserve mention. First, we did not have a comparison group to determine the impact of the intervention, and the sample size we used would have been too small to make such comparisons. Second, only 1 week separated the delivery of the modules rather than 1 month, as planned for the trial.

We conclude that context-specific infant feeding messages can be feasibly delivered by VHWs and can result in improved nutrient intakes among rural Zimbabwean infants.

## Supplementary Data

Supplementary materials are available at *Clinical Infectious Diseases* online (http://cid.oxfordjournals.org). Supplementary materials consist of data provided by the author that are published to benefit the reader. The posted materials are not copyedited. The contents of all supplementary data are the sole responsibility of the authors. Questions or messages regarding errors should be addressed to the author.
